# Supplementary material for: Snakebites in Rural Areas of Brazil by Race: Indigenous the Most Exposed Group
Source: Int J Environ Res Public Health. 2021 Sep 5;18(17):9365. doi: 10.3390/ijerph18179365 (PMC8431164; doi:10.3390/ijerph18179365)
Supplement: Supplementary file 1 [file ijerph-18-09365-s001.zip › ijerph-1344233-supplementary.pdf]

Supplementary Materials

Table S1. Country and region profiles, total population, percentage of population living in rural areas, literacy rate (as % of population 15 years and older), GDP per capita (Reais), number of hospitals with antivenom, Brazil

Region	Total population[a]	Pop living in rural areas (%)[a]	Literacy rate (%)[b]	GDP per capita (Reais, 2009)[c]	No of hospitals with antivenom [d]
North	15,864,454	26.5	90.9	17,213	292
Northeast	53,081,950	26.8	83.8	12,955	275
Southeast	80,364,410	7.1	95.7	34,790	343
South	27,386,891	15.1	95.9	30,495	578
Central- West	14,058,094	11.2	94.3	32,826	118
Brazil	190,755,799	15.6	92.0	26,446	1606

Sources and legend:

[a] IBGE (2010)

[b] IBGE (2019)

[c] IBGE (2016)

[d] Ministry of Health of Brazil (circa 2019)

Table S2. Case classification by genus of the snake according to the Ministry of Health guideline and the number of ampoules recommended (non-official translation)

Accident	Antivenom	Severity	Number of ampoules
**Bothropic**	**SAB[b] SABL[c] or SABC[d]**	Mild: Non-evident symptoms, i.e., light bleeding in the skin and/or mucous membranes; there may only be a clotting issue	**2 - 4**
		Moderate: Evident edema and ecchymosis, bleeding without compromising the general condition; there may be a clotting issue	**4 - 8**
		Serious: Severe local changes, severe hemorrhage, hypotension/shock, renal failure, anuria; there may be a clotting issue	**12**
**Lachetic[a]**	**SABL**	Moderate: Evident symptoms; there may be bleeding, no vagal manifestations	**10**
		Serious: Intense localized symptoms upon presentation, intense hemorrhage, with vagal manifestations	**20**
**Crotalic**	**SAC[e] or SABC**	Mild: Mild neuroparalytic changes; no myalgia, darkening of urine, or oliguria	**5**

		Moderate: Evident neuroparalytic changes, mild myalgia and myoglobinuria (dark urine)	**10**
		Serious: Evident neuroparalytic alterations, intense myalgia and myoglobinuria, oliguria	**20**
**Elapid**	**SAEla[f]**	Consider all cases as potentially serious due to the risk of insufficient respiration	**10**

Source: Adapted from the manual for the diagnosis and treatment of accidents by venomous animals (2001) and the epidemiological surveillance guide (2009).

Lachetic[a] = due to the potential seriousness of a lachetic envenomation accident, they are always considered clinically moderate or severe, with no mild cases

SAB[b] = Antibothopic serum (pentavalent)

SABL[c] = Antibothopic (pentavalent) and antilachetic serum

SABC[d] = Antibothopic (pentavalent) and anticrotalytic serum

SAC[e] = Anticrotalytic serum

SAEla[f] = Antielapedic serum (bivant)

Table S3. Cases of snakebite with reported information on the genus of the snake, in rural population, by state, Brazil, 2017

State	*Bothrops*	*Crotalus*	*Micrurus*	*Lachesis*	Non-venomous	Ignored	Total
Rondônia	244	1	4	5	27	21	302
Acre	188	2	2	33	4	39	268
Amazonas	883	1	4	154	35	34	1,111
Roraima	251	42	2	11	3	13	322
Pará	3,278	28	3	96	27	95	3,527
Amapá	214	3	1	17	0	13	248
Tocantins	213	55	2	2	8	33	313
Maranhão	516	216	7	6	31	68	844
Piauí	67	39	7	0	19	45	177
Ceará	357	48	14	3	59	89	570
Rio Grande do Norte	152	16	8	0	10	33	219
Paraíba	153	17	9	0	43	37	259
Pernambuco	172	53	14	4	72	140	455
Alagoas	57	26	3	2	31	31	150
Sergipe	40	5	0	0	7	26	78
Bahia	1,232	86	11	6	71	288	1,694
Minas Gerais	1,186	333	5	3	79	169	1,775
Espírito Santo	474	6	0	0	27	51	558
Rio de Janeiro	208	9	1	2	3	14	237
São Paulo	518	99	5	1	55	108	786
Paraná	337	66	2	0	80	44	529
Santa Catarina	219	4	3	0	18	24	268
Rio Grande do Sul	474	8	2	0	33	58	575
Mato Grosso do Sul	234	28	3	0	18	28	311
Mato Grosso	580	22	2	8	19	42	673
Goiás	390	80	3	0	15	34	522
Distrito Federal	24	4	1	0	1	4	34
**Brazil**	12,661	1,297	118	353	795	1,581	16,805

Figure S1. Flowchart of the study design with the number of observations of snakebites in each category, Brazil, 2017

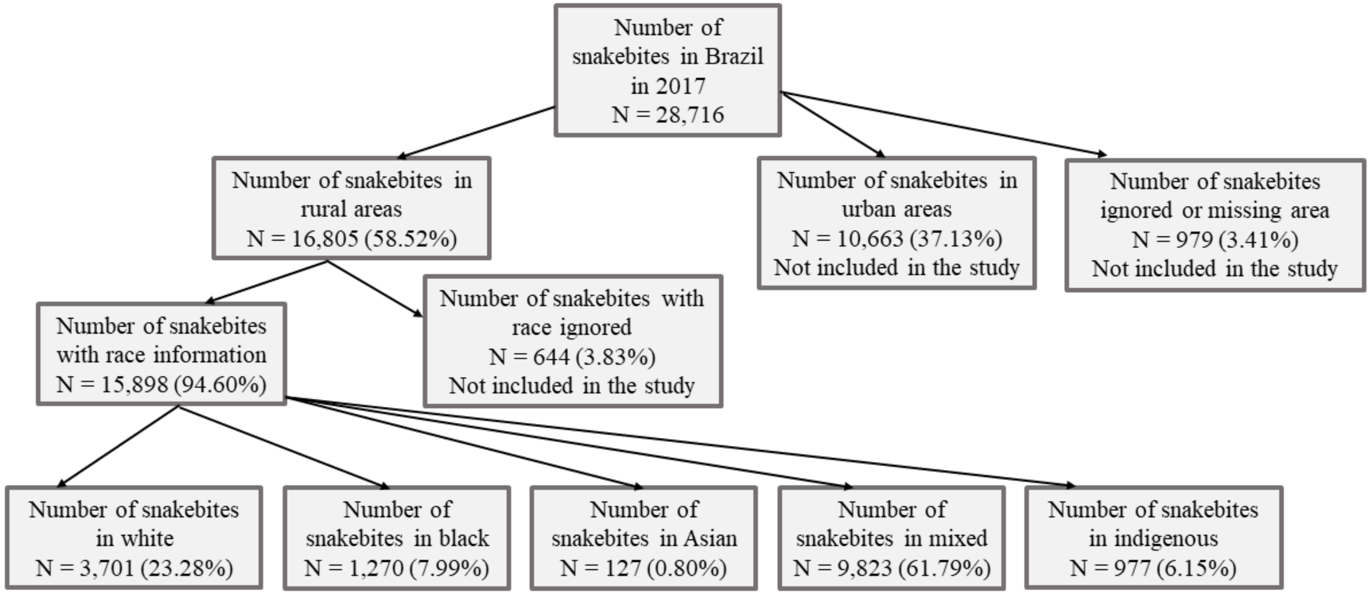

Table S4. Snakebite cases in total and rural population by race, by state and by region, Brazil, 2017.

	Cases total population							Cases rural population					
State	White	Black	Asian	Mixed	Indigenous	Unknown	Total	White	Black	Asian	Mixed	Indigenous	Total
Rondônia	96	24	3	311	15	29	478	68	16		190	13	287
Acre	35	15	1	370	22	4	447	18	9	1	218	18	264
Amazonas	42	36	8	1151	329	25	1591	23	27	6	757	285	1098
Roraima	11	10		132	277	32	462	6	8		45	244	303
Pará	287	405	36	3883	60	178	4849	199	278	28	2867	48	3420
Amapá	42	48	2	255	18	36	401	20	28	1	152	16	217
Tocantins	54	37	8	514	25	18	656	21	15	8	237	22	303
Maranhão	91	123	15	1042	69	38	1378	38	75	10	638	64	825
Piauí	20	13		223	1	17	274	14	8		145	1	168
Ceará	85	51	6	644	4	28	818	71	40	3	435	1	550
Rio Grande do Norte	57	10	1	302		55	425	33	3		147		183
Paraíba	62	27	3	253	1	70	416	34	18	1	172	1	226
Pernambuco	69	28	13	514	7	203	834	41	19	9	318	7	394
Alagoas	15	15	2	252	1	45	330	5	8		113	1	127
Sergipe	5	10	1	97		46	159	3	6		48		57
Bahia	191	401	16	1660	28	330	2626	137	283	10	1081	23	1534
Minas Gerais	1151	331	42	1630	12	184	3350	579	178	22	923	8	1710
Espírito Santo	375	62	6	250	1	44	738	302	43	5	175	1	526
Rio de Janeiro	232	78	1	142	1	156	610	109	31	1	66	1	208
São Paulo	1301	127	16	571	16	174	2205	466	55	4	202	8	735
Paraná	704	23	6	152	11	36	932	413	18	2	67	9	509
Santa Catarina	620	9	5	39	9	13	695	242		3	13	9	267
Rio Grande do Sul	772	23	2	62	18	35	912	494	11	1	34	18	558
Mato Grosso do Sul	186	22	8	243	96	29	584	89	9	1	116	83	298
Mato Grosso	335	77	9	582	104	69	1176	165	45	5	320	96	631
Goiás	267	72	11	812	4	102	1268	105	38	6	336		485
Distrito Federal	9	2		23	1	67	102	6	1		8		15

**Brazil**	7114	2079	221	16109	1130	2063	28716	3701	1270	127	9823	977	15898

Table S5. Rural population by race, snakebite cases by race, by state and by region, Brazil, 2017

	Rural Population							Cases					
States	White	Black	Asian	Mixed	Indigenous	Unknown	Total	White	Black	Asian	Mixed	Indigenous	Total
Rondônia	142646	26937	4085	230350	9109	102	413229	68	16	0	190	13	287
Acre	37555	12938	3119	134338	13326	4	201280	18	9	1	218	18	264
Amazonas	79921	30694	4155	479347	134378	0	728495	23	27	6	757	285	1098
Roraima	10683	4281	637	48594	41425	0	105620	6	8	0	45	244	303
Pará	400876	171422	18838	1769007	29115	234	2389492	199	278	28	2867	48	3420
Amapá	9816	6634	789	45201	6048	2	68490	20	28	1	152	16	217
Tocantins	54382	27501	4328	196176	10952	0	293339	21	15	8	237	22	303
North	*735879*	*280407*	*35951*	*2903013*	*244353*	*342*	*4199945*	*355*	*381*	*44*	*4466*	*646*	*5892*
Maranhão	442286	225868	25915	1705199	28361	11	2427640	38	75	10	638	64	825
Piauí	221084	99409	23941	722380	587	0	1067401	14	8	0	145	1	168
Ceará	559042	92386	22337	1425317	6726	4	2105812	71	40	3	435	1	550
Rio Grande do Norte	233318	33646	6313	428958	517	284	703036	33	3	0	147	0	183
Paraíba	328499	44663	11436	533521	9724	7	927850	34	18	1	172	1	226
Pernambuco	536969	91404	15550	1072810	27504	1	1744238	41	19	9	318	7	394
Alagoas	224324	47886	9807	532338	8276	3	822634	5	8	0	113	1	127
Sergipe	129817	41607	5627	369813	773	14	547651	3	6	0	48	0	57
Bahia	829673	515301	39592	2509708	20061	95	3914430	137	283	10	1081	23	1534

Northeast	*3505012*	*1192170*	*160518*	*9300044*	*102529*	*419*	*14260692*	*376*	*460*	*33*	*3097*	*98*	*4064*
Minas Gerais	1166095	225194	20263	1459290	11269	3	2882114	579	178	22	923	8	1710
Espírito Santo	282933	41372	2390	253974	2811	0	583480	302	43	5	175	1	526
Rio de Janeiro	244843	70256	3995	205847	738	11	525690	109	31	1	66	1	208
São Paulo	1073471	69939	19085	510122	3879	452	1676948	466	55	4	202	8	735
Southeast	*2767342*	*406761*	*45733*	*2429233*	*18697*	*466*	*5668232*	*1456*	*307*	*32*	*1366*	*18*	*3179*
Paraná	1061244	39284	11799	406096	13406	5	1531834	413	18	2	67	9	509
Santa Catarina	844120	23772	3768	120499	8361	3	1000523	242	0	3	13	9	267
Rio Grande do Sul	1363109	51535	7389	152428	19169	8	1593638	494	11	1	34	18	558
South	*3268473*	*114591*	*22956*	*679023*	*40936*	*16*	*4125995*	*1149*	*29*	*6*	*114*	*36*	*1334*
Mato Grosso do Sul	126864	14027	2179	149877	58838	1	351786	89	9	1	116	83	298
Mato Grosso	183165	33452	4221	294746	36717	20	552321	165	45	5	320	96	631
Goiás	223141	39123	7654	312641	514	1	583074	105	38	6	336	0	485
Distrito Federal	29241	6961	1699	49832	187	30	87950	6	1	0	8	0	15
Central-West	*562411*	*93563*	*15753*	*807096*	*96256*	*52*	*1575131*	*365*	*93*	*12*	*780*	*179*	*1429*
**Brazil**	10839117	2087492	280911	16118409	502771	1295	29829995	3701	1270	127	9823	977	15898
